# Combined Phacoemulsification and Intravitreal Dexamethasone Is an Effective Option for High-Risk Diabetic Macula Oedema Patients

**DOI:** 10.7759/cureus.17603

**Published:** 2021-08-31

**Authors:** Arun J Thirunavukarasu, Andrew Malem, Spyridon Mourtzoukos

**Affiliations:** 1 Department of Ophthalmology, Cambridge University Hospitals NHS Foundation Trust, Cambridge, GBR; 2 Department of Ophthalmology, School of Clinical Medicine, University of Cambridge, Cambridge, GBR; 3 Department of Ophthalmology, Corpus Christi College, University of Cambridge, Cambridge, GBR; 4 Department of Ophthalmology, Queen Alexandra Hospital, Portsmouth, GBR

**Keywords:** diabetic macular oedema, cataract surgery, intraoperative dexamethasone, phacoemulsification, ozurdex, diabetic retinopathy, diabetes, central macular thickness, steroids

## Abstract

Objective

Cataract surgery in diabetic patients carries an increased risk of post-operative macula oedema, particularly in those with a history of diabetic macula oedema (DMO) treatment or DMO at the time of surgery. We investigated whether simultaneous phacoemulsification with intravitreal Ozurdex^®^ reduces the risk of developing new, or deteriorating current, DMO.

Methods

We conducted a retrospective review of 79 consecutive ‘high-risk’ diabetic patients who underwent phacoemulsification with intraocular lens insertion and intravitreal Ozurdex^®^ implantation immediately subsequently. ‘High risk’ was defined as diabetic patients with prior treatment history for DMO or current DMO. Central macula thickness (CMT), best-corrected visual acuity and intraocular pressure were recorded pre-operatively, at two to four weeks and at three months post-operatively. A significant change in CMT was defined as a change of ≥0.1 LogOCT units.

Results

The mean age was 72.6 years; 52% were males. The mean pre-operative CMT was 365um. Thirty-seven per cent (37%) patients had prior DMO history that had resolved; 63% had confirmed DMO in surgery. Two to four weeks post-operatively, 82% of patients had stable CMT and 18% showed improvement. No patients deteriorated. Three months post-operatively, 48% of patients had stable CMT relative to pre-operative measurements, 38% improved, and 14% deteriorated. Analysis of variance (ANOVA) indicated no significant differences in response with demographical or pathological factors, including diabetic retinopathy grade and treatment history.

Conclusion

Phacoemulsification surgery combined with Ozurdex^®^ insertion at the end of the procedure is a highly effective strategy for protecting against the formation of new, or the deterioration of current DMO, in the highest risk diabetic patients undergoing cataract surgery.

## Introduction

Over 470 million people are affected by diabetes globally, with this number expected to increase to 570 million by 2025 [1]. It is therefore not surprising that diabetes is the third most common co-morbidity for patients undergoing cataract surgery [2]. Diabetic patients are known to develop cataracts earlier than non-diabetics with surgery carrying higher risks of intra and post-operative complications and poorer visual outcomes [3-7]. Of particular importance is the increased risk of post-operative diabetic macula oedema (DMO), which has a tendency to be difficult to treat and persist [4]. In diabetic patients with no history of DMO, rates of new DMO following surgery range between 20% and 30% [4,8-10]. This risk is higher in patients who have a history of previous DMO or who have chronic DMO at the time of surgery [11-12].

Deciding when to perform cataract surgery on these high-risk patients is controversial. The optimal treatment strategy for these particularly high-risk patients has been the subject of much debate, with a variety of treatment protocols being advocated [13]. These vary from withholding cataract surgery until the macula is completely dry, which is sometimes impossible, to the use of anti-vascular endothelial growth factor (anti-VEGF) in the immediate pre-operative period. Concomitant use of triamcinolone has also been studied previously, with promising results [14]. In the UK, the National Institute for Clinical Excellence (NICE) guidance and approval for intravitreal dexamethasone (Ozurdex®) use relies on the patient being pseudophakic.

In this retrospective study, we assessed results from the highest risk diabetic patients who underwent simultaneous cataract surgery and intravitreal Ozurdex® dexamethasone implantation. All patients received intravitreous Ozurdex® immediately following the completion of uncomplicated phacoemulsification surgery. The rationale for using concomitant intravitreal Ozurdex® is the reported correlation between aqueous inflammatory and angiogenic cytokine levels and post-operative macular oedema [15-16].

## Materials and methods

We performed a retrospective review of 79 consecutive high-risk diabetic patients who underwent simultaneous cataract extraction with intravitreal Ozurdex® (Allergan plc, Dublin, Ireland) implantation between December 2015 and February 2019. All phacoemulsification procedures were performed by the same consultant at a single unit and a posterior chamber intraocular lens was inserted in the capsular bag in each case. Immediately following insertion of the lens, an intravitreal Ozurdex® was implanted via a tunnelled injection 3.5 mm from the limbus. All patients received post-operative chloramphenicol drops four times per day but no topical steroids. As data were analysed retrospectively, the study was exempt from ethics approval, although all procedures adhered to the tenets of the Declaration of Helsinki, and patients gave written, informed consent prior to surgery. Inclusion criteria for the ‘high-risk’ cohort consisted of a diagnosis of diabetes and a history of either previously treated DMO or current DMO at the time of cataract surgery.

Baseline subject characteristics recorded for all patients consisted of age, gender, duration of diabetes, diabetes type (1 or 2), diabetic retinopathy grade, and method of diabetes control (insulin dependence vs diet/tablet control). Any previous treatment with macula laser or anti-VEGF/triamcinolone was also recorded as well as best-corrected visual acuity (BCVA), intraocular pressure (IOP), history of a previous vitrectomy and the presence of an epiretinal membrane or vitreomacular traction (VMT).

Prior to listing for surgery, all patients had undergone a baseline dilated fundal examination by a diabetic retinal consultant and the diabetic grade was recorded according to the National Retinopathy Screening screening grading system. Pre-operative spectral-domain optical coherence topography (SD-OCT) (Heidelberg, Germany) was performed four weeks prior to surgery in all patients. The central macula thickness (CMT) was recorded by a trained technician. Follow-up reviews were performed at two to four weeks and three months after surgery. At these reviews, repeat macula SD-OCT scans were performed and macula CMT recorded as well as IOP and BCVA. Due to the wide variation in the literature in reporting significant changes in CMT [17-18], we defined a significant change in CMT as a change of 0.1 LogOCT units or move [19].

LogOCT is calculated as log10[CMT/200] and offers a series of benefits over the traditional simple comparison of absolute CMT values over time. A change in 0.1 LogOCT has been postulated to more closely correlate to real-world clinical changes in visual function and acuity (Ferris et al., 2010). Furthermore, the absolute value of a 0.1LogOCT unit change varies with individual patients’ baseline CMT, with a thicker baseline CMT requiring a greater absolute change in CMT to reach the 0.1 LogOCT unit threshold. Unlike using absolute CMT changes (e.g. of 50µm), LogOCT is individualised, better reflecting relative changes. A change of 0.1 LogOCT units is also beyond the intrinsic noise of OCT testing, again aiding the detection of a ‘real’ change. Finally, taking LogOCT introduces a normalising transformation to the data, facilitating parametric statistical analysis.

## Results

Summary statistics for the cohort (N = 79) are presented in Table 1.

**Table 1:** Summary statistics Summary statistics for the cohort of high-risk diabetic macular oedema patients requiring cataract surgery, detailing demographics and relevant past medical history. VEGF: vascular endothelial growth factor

Table 1	N = 79
Age in years:	
Mean (SD)	72.6 (9.3)
Median (minimum, maximum)	75 (39, 94)
1^st^ quartile, 3^rd^ quartile	68.5, 80
Sex:	
Male	41 (52%)
Female	38 (48%)
Eye:	
Left	46 (58%)
Right	33 (42%)
Diabetic macular oedema status:	
Previously treated, resolved at the time of surgery	29 (37%)
Active at the time of surgery	50 (63%)
Tractional element status:	
Epiretinal membrane	7 (8.9%)
Vitreoretinal traction	3 (3.8%)
None	69 (87%)
Previous laser treatment history:	
Yes	25 (32%)
No	54 (68%)
Previous injection history:	
None	43 (54%)
Anti-VEGF	32 (41%)
Steroid	2 (2.5%)
Anti-VEGF and steroid	2 (2.5%)

Parametric analysis was justified for logOCT data as distributions appeared normal in graphical presentation (density plots, histograms, and quantile-quantile plots) and did not exhibit statistically significant deviation in Shapiro-Wilks tests for the first two time periods, with only a small deviation at the third time period (Table 2) unlike CMT data. The slight deviation from normality three months post-operatively was offset by the significant sample size, which compensates according to the central limits theorem. BCVA was not normally distributed at any time period, according to graphed distributions or Shapiro-Wilks tests (Table 2), contraindicating parametric testing.

**Table 2:** Testing normal distribution Shapiro-Wilks tests for central macular thickness (CMT), logOCT and best-corrected visual acuity (BCVA), measured at three time-points relative to surgery

Table 2	Time	W	p
CMT	Pre-operative	0.956	8.1E-03
CMT	2-4 weeks post-operative	0.914	2.6E-04
CMT	3 months post-operative	0.890	7.6E-06
logOCT	Pre-operative	0.983	3.9E-01
logOCT	2-4 weeks post-operative	0.974	1.9E-01
logOCT	3 months post-operative	0.968	4.9E-02
BCVA	Pre-operative	0.706	3.3E-11
BCVA	2-4 weeks post-operative	0.852	3.9E-07
BCVA	3 months post-operative	0.852	4.9E-06

**Figure 1:**
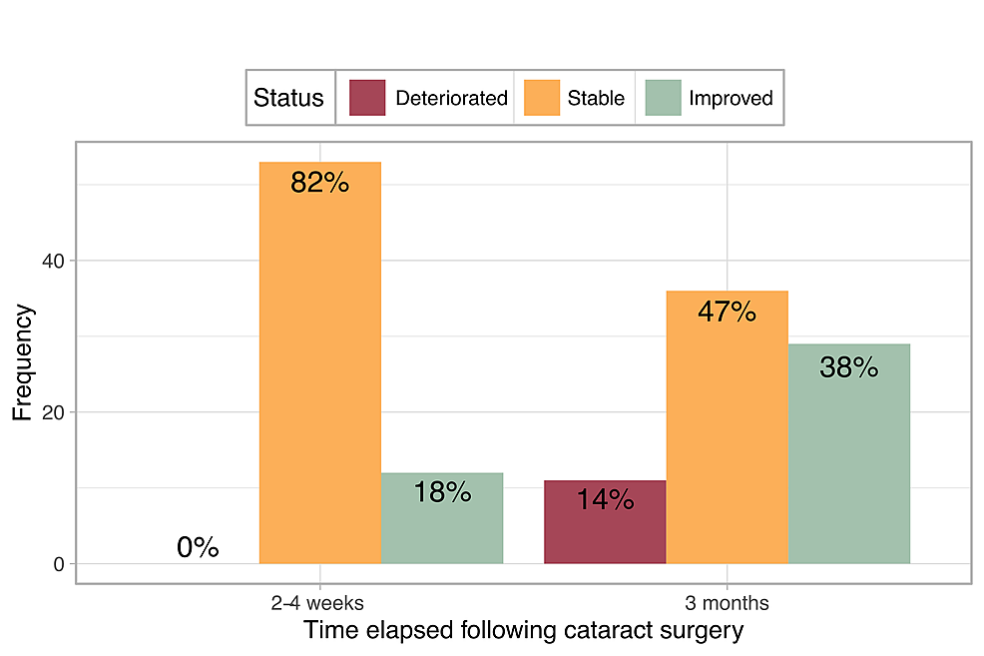
DMO change following surgery Changes in the diabetic macular oedema of patients before, as well as two to four weeks and three months post-surgery. Significant changes in central macular thickness were defined as greater than 0.1 logOCT increase or decrease. No deterioration was observed at the two to four-week follow-up. DMO: diabetic macula oedema

LogOCT changes were defined as a deterioration if CMT increased by 0.1 LogOCT units or more relative to pre-operative levels. An improvement was defined as a reduction of CMT by 0.1 LogOCT units or more. At two to four weeks post-operatively, 82% of patients had stable CMT measurements and 18% improved. No patients exhibited deterioration. At three months post-operatively, 47% remained stable, 38% showed an improvement in CMT levels relative to pre-operative measurements and 14% deteriorated (Figure 1).

**Figure 2:**
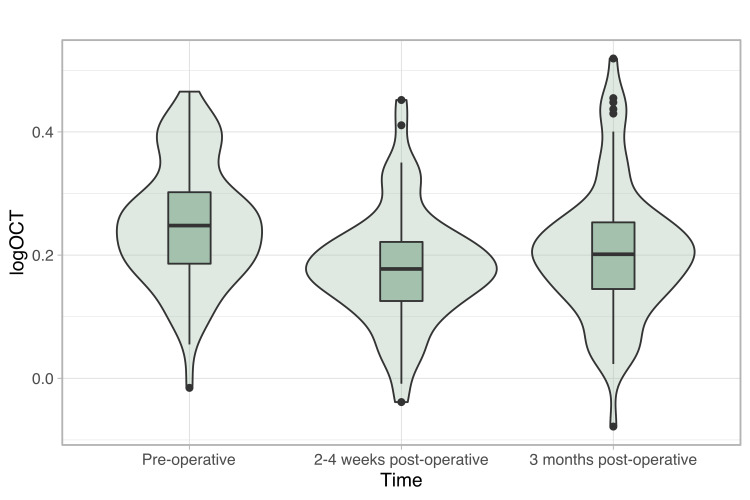
LogOCT across time LogOCT, as measured at the three described time-points before and after surgery. Central macular thickness improved two to four weeks after surgery, and improvement was maintained two to four months later.

One-way analysis of variance (ANOVA) results indicated that significant changes in logOCT (distributions depicted in Figure 2) occurred following surgery: F(2,217) = 9.16, p = 1.5x10-4. Post-hoc t-tests with Benjamini-Hochberg correction applied revealed logOCT was significantly lower (i.e. improved) two to four weeks (p = 1.4x10-4) and three months (p = 7.3x10-3) post-operatively than pre-operatively.

With Welch t-tests, changes in logOCT were found not to differ between patient groups with and without a prior history of resolved DMO (p2-4w = 0.38, p3m = 0.42), presence of a tractional element at the time of surgery (p2-4w = 0.57, p3m = 0.20), or prior laser treatment (p2-4w = 0.47, p3m = 0.18), two to four weeks and three months post-operatively, respectively. No significant difference was observed after grouping by sex (p2-4w = 0.98, p3m = 0.76) or eye side (p2-4w = 0.35, p3m = 0.56). ANOVAs revealed no significant difference between groups with a history of prior steroid and/or anti-VEGF injections or not at two to four weeks (F(2,62)=0.106, p = 0.90) and three months (F(3,72) = 1.743, p = 0.17) post-operatively. There was no significant association between change in LogOCT and age (Pearson’s correlation coefficient, ρ2-4w = 0.07, p2-4w = 0.57, ρ3m = 0.04, p3m = 0.68).

**Figure 3:**
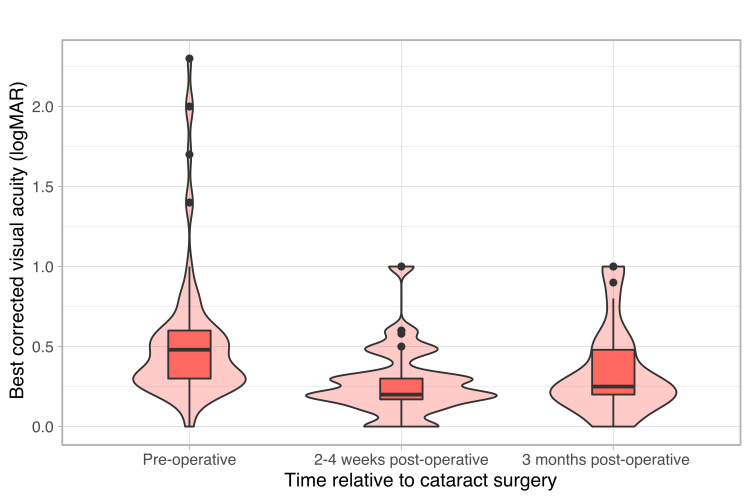
Best-corrected visual acuity across time Best-corrected visual acuity measured across time in the diabetic macular oedema cohort; before, two to four weeks and three months after cataract surgery. Acuity improved following cataract surgery, with improvement maintained three months post-operatively.

Visual acuity (VA) improved similarly consistently post-operatively (Figure 3), with significant Kruskal-Wallis result (H(2) = 33, p = 6.6x10-8), the Welsh t-test confirmed improvement at two to four weeks (p = 6.9x10-8) and three months (p = 1.7x10-4) after surgery. Correlation between VA and logOCT indicated that a component of the observed maintained improvement was due to controlled DMO (Pearson’s correlation coefficient, ρ = 0.25, p = 0.0003).

The mean change in IOP two to four weeks post-operatively was -0.207mmHg (σ = 3.8), and three months post-operatively was -0.882 mmHg (σ = 4.5) - a decrease at both timepoints. The mean change between two to four weeks and three months was a further decrease: -0.88 mmHg (σ = 4.5). The Kruskal-Wallis test indicated no significant difference at any of the three time points: H(2) = 0.169, p = 0.64. One patient had ocular hypertension (IOP > 21 mmHg) before surgery; three patients had ocular hypertension two to four weeks post-operatively; zero patients had ocular hypertension three months post-operatively and none required any topical ocular hypotensive treatment.

## Discussion

There is a growing evidence base for the use of intraoperative Ozurdex® in diabetic patients requiring cataract surgery. Many studies are small in scale [20-21], with resultant limited power making a conclusion on efficacy and risk more challenging, although positive conclusions are consistent, including in larger studies [18]. In this study, the vast majority of patients either improved or remained stable in terms of CMT over the three months post-operative monitoring period. At the end of the active period of intravitreal Ozurdex®, a small proportion (14%) did deteriorate again and therefore ongoing monitoring and treatment is required in these high-risk patients; it is likely that diabetes-associated impairment of the blood-aqueous barrier prolongs inflammation and oedema risk [22-23]. BCVA improved similarly consistently, and IOP did not tend to increase significantly, perhaps surprisingly given the documented risk associated with corticosteroid treatment [24].

High-risk diabetic patients respond equally well with combined treatment irrespective of the patients age, sex, duration of diabetes, type of diabetes, previous treatment for DMO, presence of vitreomacular pathology and grade of diabetes, and our analysis revealed no common factor linking the minority of individuals that did exhibit deterioration. Together, this suggests that the combined treatment strategy could be offered to all high-risk patients. These patients have traditionally had to wait longer than optimal for cataract surgery and have previously been shown to have significantly poorer post-operative visual gains - with this combined approach, this does not have to be the case.

Our study was limited by its retrospective design; randomised control trials in the described high-risk population are complicated by the lack of alternative treatments; patients tend not to receive surgery instead, especially when significant DMO is present and anti-VEGF therapy has been unsuccessful. Future investigations could seek to establish fair control groups to compare to high-risk diabetic macular oedema patients receiving our described combined treatment, in order to conduct a randomised, prospective study of efficacy and safety.

## Conclusions

A combined phacoemulsification/Ozurdex® therapeutic strategy is effective in even the most challenging patient cases, complicated by the higher risks associated with prior or current DMO. This study adds to the evidence base regarding intraoperative dexamethasone implantation, in a uniquely specific cohort of high-risk patients. In addition, this study exemplifies a means of defining significant changes in DMO, which could improve the quality of future investigations, and make meta-analysis more feasible. Further investigation is required to a) verify the efficacy of intraoperative dexamethasone implantation in higher risk cohorts; b) determine when it is beneficial to offer intravitreal dexamethasone with cataract surgery as opposed to alternative treatment plans (or no treatment); c) produce fair criteria justifying the cost of intraoperative dexamethasone, including for the minority of higher-risk patients.
